# Feasibility and acceptability of a nursing intervention with family caregiver on self-care among heart failure patients: a randomized pilot trial

**DOI:** 10.1186/s40814-016-0077-8

**Published:** 2016-07-21

**Authors:** Sylvie Cossette, Hayet Belaid, Sonia Heppell, Tanya Mailhot, Marie-Claude Guertin

**Affiliations:** 1 Faculty of Nursing, University of Montreal, Montreal Heart Institute Research Center S-2510, 5000 Belanger Street, Montreal, Quebec H1T 1C8 Canada; 2 Heart Failure Clinic, Montreal Heart Institute Research Center S-2490, 5000 Belanger Street, Montreal, Quebec H1T 1C8 Canada; 3 Montreal Heart Institute, 5000 Belanger Street, Montreal, Quebec H1T 1C8 Canada; 4 Montreal Health Innovations Coordinating Center, Suite 400, 4100 Rue Molson, Montréal, Québec H1Y 3N1 Canada

**Keywords:** Heart failure, Self-care, Family caregiver, Self-Determination Theory, Nursing intervention

## Abstract

**Background:**

Self-care practices in heart failure (HF) contribute to quality of life, symptom stabilization, and extended life expectancy. However, adherence to practices such as liquid and salt restriction or symptom monitoring require high motivation on a daily basis. The aim was to assess the feasibility, acceptability, and potential effectiveness of a nursing intervention with family caregivers, aimed at improving self-care practice of HF patients.

**Methods:**

This pilot study involved 32 HF patient-caregiver dyads (16/group) randomized to an experimental (EG) or control group (CG). The intervention, based on the Self-Determination Theory, was designed to enhance patients’ autonomy and motivation in self-care practices, by involving their caregivers’ support. Five encounters were planned with the EG dyads—two face-to-face during hospitalization and three by telephone after discharge. The feasibility of delivering the protocol was evaluated as well as the acceptability of the intervention. The potential effectiveness of the intervention was assessed based on patient outcomes, including general self-care management and self-care specific to HF, perceived competence to manage HF, autonomous motivation (A-motivation, external extrinsic motivation, internal extrinsic motivation, and intrinsic motivation), and perceived support from the caregiver. Caregiver outcomes included level of support provided to the patient.

**Results:**

Despite recruitment challenges, the intervention was feasible, with 12 of the 16 dyads receiving all 5 encounters delivered per protocol. The 4 other dyads received the two hospital encounters, but at least 1 of the 3 post-discharge planned telephone encounters was not feasible because the patients had been re-hospitalized or was deceased. Participant’s satisfaction with the intervention was high. Outcomes favoring the EG include self-care specific to HF, internal extrinsic motivation, intrinsic motivation, and caregiver’s feeling that they provide a higher level of support.

**Conclusions:**

Caregiver involvement was found to be both a feasible and acceptable means of supporting self-care practice in HF patients. This approach presents a potential avenue for enhancing patients’ efforts in this regard. However, this pilot study offers preliminary findings only, which need to be replicated in a phase 3 clinical trial.

## Background

Heart failure (HF) is a chronic disease with severe symptoms such as breathlessness and fatigue that can seriously affect patients’ and families’ quality of life [1]. The success of pharmacological and non-pharmacological symptom management depends on patient adherence to several self-care practices, such as a salt and fluid restriction, regular weight monitoring, medication adherence, physical activity, and self-monitoring of symptoms [2]. As previously defined by Orem, self-care refers to the practices of specific deliberate activities undertaken by individuals that maintain their integrity, as well as their development [3]. Poor adherence to these self-care practices is strongly related to reduced quality of life, re-hospitalization, and reduced life expectancy [4].

Factors related to patients’ poor self-care practices include lack of specific knowledge and skills, misconceptions about heart failure, psychological factors as well as lack of social support and motivation [5, 6]. A systematic review of qualitative studies suggest that one of the main contextual factors that influence patient’s self-care practices includes caregivers’ support [7]. According to this review, caregivers are mostly involved in facilitating three aspects of self-care pratices: medication, sodium reduction, and symptom recognition. Clinical guidelines recognized the need for education of HF patients and the development of a plan of action to manage symptoms and adhere to self-care practice including the prescribed treatments [4]. To attain these goals, knowledge and skills must grow from autonomy and motivation rather than from fear messaging, criticism, and autocracy [8].

The inclusion of a caregiver in the care process can facilitate patient adherence to self-care confidence if the caregiver emphasizes autonomy and motivation rather than dependence and criticism [4]. In a correlational study, it was observed that caregiver support has an effect on patients’ treatment adherence and implementation of behaviors to manage HF and its symptoms [9]. Caregiver support may positively influence self-care confidence which in turn will result in better self-care behaviors [10]. For instance, an experimental study [11] demonstrated that involving a caregiver as a partner to provide supportive interventions improved HF patients’ reduction in sodium intake. However, as reported by Clark et al. [2], caregivers may not always contribute positively to self-care management despite their willingness to help, for example, by overprotecting the person with HF.

The intervention tested by Dunbar and colleagues [11] was based on the Self-Determination Theory (SDT) [12] which promotes autonomy in the practice of self-care and suggests that three basic needs—perceived competence, autonomous motivation, and perceived relatedness—determine human behaviors. Fulfilling these needs using appropriate interventions can help patients internalize their motivation to adopt self-care practices, so that it emanates from within rather than from outside pressure [12].

The first need, “perceived competence” to perform self-care, can be enhanced by educating patients and providing information, as well as offering opportunities to practice—for instance, calculating liquid and salt ingestion.

The second need, “autonomous motivation”, refers to the desire to accomplish self-care activities for oneself, rather than at the request of others. Degrees of autonomy range on a continuum from externally to internally regulated. In externally regulated autonomy, patients are A-motivated, i.e., not motivated to perform self-care because they see no positive benefit and comply only because of external demands. On the other hand, patients with internal motivation recognize the benefits of self-care and are eager to adopt and persevere in self-care practices. Internally regulated motivation may be enhanced by nursing interventions, for instance, by offering empathy and choices rather than imposing rules, providing positive feedback rather than criticizing, and focusing on problem-solving rather than blaming the patient [6].

The third need, “perceived relatedness”, is a key factor in the present study. Relatedness refers to a universal human need to connect with and to be supported by others [12]. For instance, adhering to a low-salt diet would be easier if the patient’s autonomy to adhere to this practice is promoted and encouraged in the home environment. Thus, supportive attitudes from caregivers may enhance a patient’s perception of relatedness, which in turn is thought to enhance self-care practices.

In sum, involving caregivers with a nursing intervention based on SDT principles to support HF patients’ autonomy could be an avenue towards enhancing their motivation for performing self-care practices.

### Purpose

The goal of this randomized pilot study was to assess the feasibility, acceptability, and potential effectiveness of a nursing intervention with caregivers, aimed at improving self-care practices of HF patients. We also evaluated the potential effectiveness on outcomes which were expected to favor the experimental group patients when compared to the control group patients: higher general self-care management (H1); higher self-care specific to HF (H2); higher perceived competence to manage HF (H3); autonomous motivation reflected by lower A-motivation (H4_a_), lower external extrinsic motivation (H4_b_), higher internal extrinsic motivation (H4_c_), and higher intrinsic motivation (H4_d_); and higher perceived support from the caregiver (H5). Additionally, potential effectiveness was assessed based on caregivers’ perceived level of support to the patient—as we expected the level of support to be higher in the experimental group than that in the control group (H6).

## Methods

### Design

We designed a randomized experimental pilot study to compare our experimental intervention with usual care. Table 1 illustrates the study design and timeline.

**Table 1:** Study design and timeline

Template adapted from the SPIRIT guideline									
Participants timeline		In hospital				After hospitalization			
		Enrolment and baseline measures	Randomization	Before discharge encounters		Post-discharge encounters			One-month measures
Control group	Patient and caregiver	√	√	Usual care	Usual care				√
Intervention group	Patient	√	√	Usual care and encounter #1	Usual care	Encounter #3	Encounter #4	Encounter #5	√
	Caregiver	√	√	Usual care and encounter #1	Usual care and encounter #2	Encounter #3	Encounter #4	Encounter #5	√

### Study setting and participants

The study was conducted in HF patients hospitalized in a tertiary cardiac hospital in Montreal, Canada, and their primary caregiver. To be eligible, HF patients had to return home after hospital discharge and live with a primary caregiver (either spouse, adult child, sibling, or significant other) who agreed to participate in the study. Exclusion criteria included inability to understand spoken and written French, cognitive problems (e.g., dementia) that would preclude provision of informed consent, and a planned regular specialized follow-up, for example, at a heart failure clinic or palliative care program, because these services would result in co-intervention bias.

During hospitalization, potential participants received usual care from the bedside nurse until hospital discharge. During the hospitalization, once their clinical condition had stabilized, the study was explained to eligible patients, informed consent was obtained from them and their caregivers, and baseline data were collected before randomization. Follow-up measures were collected by telephone at 1 month after hospital discharge by a research assistant blind to the study group allocation.

### Interventions

The control group (CG) received usual discharge planning and referrals but no intervention from the study nurse. Discharge planning in usual care is offered by the bedside nurses who provide information on HF, medication, and nutrition. There are no telephone or hospital visit follow-ups aside from the medical follow-up offered by the patient’s cardiologists.

For the experimental group (EG), the intervention included five encounters—two face-to-face during hospitalization and three by telephone after discharge (Table 1). The first face-to-face encounter was conducted with the dyad and the second with the caregivers only because the focus of these two encounters differed. The first encounter focused on the patient’s needs, and the second was about the caregiver’s supportive attitudes. The medical condition of an HF patient in hospital prevents him from optimal information retention, hence the presence of a caregiver to support him. As the second encounter focused on the caregiver learning how to facilitate the patient’s autonomy through role-playing, it was not appropriate to solicit the patient.

We chose telephone rather than hospital follow-up after discharge in order to both reduce cost and facilitate a future transfer to practice. The telephone encounters were conducted either with the dyad together on speaker phone or with the patient or caregiver one at a time. The protocol allowed between 30 and 45 min for each face-to-face encounter and 10 min for each phone call.

The content of the intervention was based on Deci and Ryan’s SDT [13, 14]. Patients and caregivers were involved as active partners, and the project nurse addressed the dyad as a whole to promote patient relatedness. Based on SDT principles, the project nurse interacted with the dyad by offering choice rather than imposing restrictions, avoiding criticism, encouraging empathy, and giving positive reinforcement. Patients and caregivers were encouraged to share any concerns either during or between encounters, so that all ambiguities regarding self-care activities were discussed as they occurred. Learning activities include the nurse acting as role model for the caregivers to help them adopt supportive attitudes and behaviors in their own subsequent interactions with the patient. Role-playing involving the project nurse and the caregiver were also planned during the encounters to provide practice in autonomy-supportive behaviors relating to patient self-care. For instance, in a vignette where the caregiver and patient share a buffet filled with salty dishes, the nurse guided the patient towards less salty choices instead of advising against everything.

A patient intervention checklist and caregiver intervention checklist were developed as a guide. The patient intervention checklist integrated the three basic needs proposed in the Self-Determination Theory (SDT): perceived competence, autonomous motivation, and perceived relatedness. This checklist includes 20 items, 11of which refer to “assessment” (e.g., what are you doing to manage the symptoms of HF?) and 9 refer to “interventions” (e.g., providing information to the patient on potential benefits of performing self-care activities). The caregiver checklist includes seven items describing “interventions” (e.g., exploring potential strategies with the caregiver to support HF patients without criticism). After each encounter, the project nurse checked off which assessment and nursing intervention was retained in response to the specific context of the dyad. Because the intervention was individualized, each dyad could receive a different intervention package. The checklists also served to describe the interventions delivered to both patients and caregivers as recommended in the TIDieR template [15].

The project nurse held a bachelor’s degree and 2 years of experience with HF patients. She was trained and coached during the study by her study supervisor and a nurse practitioner with extensive experience in motivational interventions for HF patients and caregivers.

### Measures

#### Feasibility and acceptability measures

The feasibility of delivering the intervention’s structure was evaluated by examining the number and timing of actual versus planned encounters and the duration of each encounter. To assess delivery of the intervention content, we used data on the patient intervention checklist and the caregiver intervention checklist that was filled out after each encounter by the project nurse. Recruitment issues were also examined as an indicator of feasibility.

The Treatment Acceptability and Preference Questionnaire (TAPQ) [16] assessed the acceptability of the intervention to EG patients and their caregivers (independently). The TAPQ includes four items assessing whether the intervention was perceived as appropriate, acceptable, and effective in helping to manage the HF and whether subjects would be willing to participate if a similar study was offered to them. Answers on a 5-point Likert scale ranged from “not at all” (0) to “extremely” (4). In addition, we added an overall question about satisfaction with the intervention. The Cronbach alpha reported by Sidani et al. [16] ranged from 0.80 to 0.87.

#### Outcome measures

Outcome measures for examining potential effectiveness were administered at baseline during the hospitalization and readministered by telephone at 1 month post-discharge. English scales were translated to French using the back-translation method defined by the World Health Organisation, with clarification and content validation of the translated items by heart failure experts, as well as pretesting with patients [17, 18].

General self-care management (H1) was assessed by the Therapeutic Self-Care Scale (TSCS) [19]. This instrument measures 12 actions taken by a patient to promote, maintain, or improve health, prevent sickness, detect and manage symptoms, and regain normal functioning. Patients indicated whether they agreed with each statement from “not at all” (0) to “totally” (4). A higher score indicates a higher independence in general self-care behaviors. Cronbach alpha ranging between 0.88 and 0.93 for the English version and 0.86 for the French version were previously reported [18, 20].

Self-care specific to HF (H2) was assessed by the Self-Care of Heart Failure Index (SCHFI) [21], Section A, version 6, for instance medication and weight monitoring. Section A includes ten items assessed on a 4-point Likert scale ranging from “rarely or never” (1) to “always or daily” (4), with a higher score representing higher frequency of self-care activities. The SCHFI subscale raw score was standardized to a scale ranging from 0 to 100 with scores above 70 reflecting adequate self-care activities [21]. Cronbach alphas of 0.55 for the English Version [22] and 0.56 and 0.54 for the French version were previously reported [17].

Perceived competence to manage HF (H3) was assessed by the Perceived Competence Scale (PCS) [13]. The PCS includes four items assessed on a 7-point Likert scale from “not true at all” (1) to “totally true” (7). Higher scores indicate higher perceptions of perceived competence. Reported Cronbach alphas were higher than 0.80 in a study in diabetic patients [14]. Since the PCS “can be easily adapted to study additional behaviors or behavioral domains” (as suggested on the Self-determination theory official Web site [13], we replaced the word “diabetes” with “cardiac heart failure” in the present study.

The original French version of the Behavioral Questionnaire of the Elderly-Health subscale [22] measures four types of motivation (H4): (1) A-motivation reflected by the statement “I don’t know, I don’t see what it does for me”; (2) external extrinsic motivation reflected by the statement “because I’m supposed to do it”; (3) internal extrinsic motivation reflected by “I choose to do it for my own good”; and (4) intrinsic motivation reflected by “for the pleasure of doing it”. Each motivation type is assessed within three health activity categories: general health seeking, diet/nutrition, and visits to the doctor. Respondents assess how each of the four types of motivation corresponds to their situation using a 7-point scale ranked from “not at all” (1) to “corresponds exactly” (7). Therefore, the possible score for each of the four motivation scores ranges from 3 to 21 (3 health activity categories * 1- to 7-point scale). Lower scores on A-motivation and external extrinsic motivation and higher scores on internal extrinsic motivation and intrinsic motivation reflect higher autonomous motivation. Reported Cronbach alphas were 0.81 for the A-motivation, 0.87 for external extrinsic motivation, 0.85 for internal extrinsic motivation, and finally 0.84 for intrinsic motivation [22].

The level of support to the patient (H5) perceived by caregivers was assessed by the Family Care Climate Questionnaire-Patient version (FCCQ-P) [23]. The FCCQ-P includes 14 items assessed on a 4-point Likert scale from “not at all” (1) to “totally” (4). Higher scores indicate higher perceptions of support. Reported Cronbach alpha was 0.89 in a previous study [23].

The caregiver’s perception of the support they provided to the patient (H6) was assessed by the Family Care Climate Questionnaire-Family version (FCCQ-F) [23]. The FCCQ is a parallel version of the FCCQ-P with the same scoring system. Reported Cronbach alpha was 0.81 in a previous study [23].

Sociodemographic and clinical characteristics were collected in the medical chart or using self-report questionnaires at baseline.

### Sample size and randomization

The pilot study was not designed for adequate statistical power but to test the feasibility, acceptability, and potential effectiveness of the intervention [24]. A sample size of 16 dyads per group (*N* = 32 dyads) was determined based on the expected rate of recruitment.

The randomization sequence was generated by an independent statistician from the Montreal Health Innovations Coordinating Center (MHICC), using the PROC PLAN procedure in SAS (SAS Institute Inc., Cary, NC, USA). The statistician provided sealed, opaque envelopes to the project nurse. After the envelope was opened and patient-caregiver dyads were randomized to the intervention or control group, neither the nurse nor the participants were blind to the study allocation.

### Data analysis

Sociodemographic and clinical variables were summarized as mean values for continuous variables and as count and percentage for categorical variables. Cronbach alphas were calculated for all the scales, except for the TAPQ. The TAPQ was completed only by the experimental group, not the control group, as it refers to the acceptability of the experimental intervention reducing the sample size by half. We examined the potential effectiveness by the direction and amplitude of the differences between groups on the outcome measures.

## Results

### Reliability of the outcome measures

Cronbach alphas were calculated for the baseline data only because we had missing data at the 1-month data collection point, hence reducing the sample size. The Cronbach alpha for the TAPQ calculated in caregivers of the EG was 0.82. It was not calculated in the patient sample of the EG because of a lack of variability: all patients responded being “extremely” satisfied for two items of the scale. For the preliminary efficacy scales, the Cronbach alphas at baseline were 0.86 for the TSCS, 0.63 for the SCHFI, 0.87 for the PCS, 0.73, 0.68, 0.43, and 0.71 for the four motivational subscales, and 0.78 and 0.70 for the FCCQ-P and FCCQ-F, respectively. Generally, Cronbach alphas were adequate, except on internal extrinsic motivation (0.43). This low Cronbach alpha may reflect low inter-item correlations between choosing to perform—for their own good—“health related activities,” “diet,” and “visiting the doctor”.

### Participant flow

**Fig. 1:**
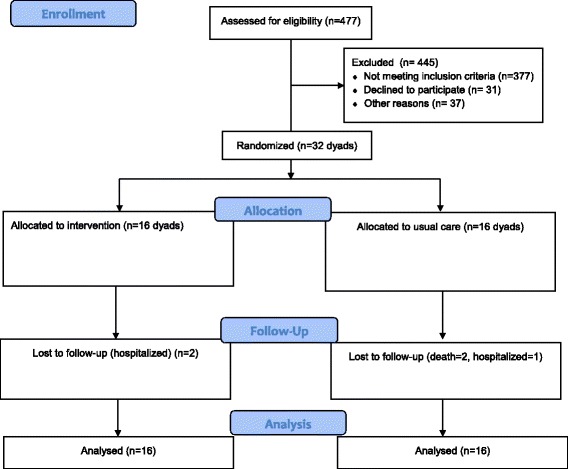
CONSORT Flow Diagram for the pilot randomized study

Recruitment started August 2010 and ended October 2011, including a 1-month follow-up (Fig. 1). The project nurse evaluated 477 potential participants, of whom 377 did not meet study inclusion criteria, mostly because they were already receiving regular follow-up (*n* = 133) or because they reported no cohabiting caregiver (*n* = 126). Another 37 patients were excluded because of logistical issues (e.g., discharge time outside of nurse’s shift), and 16 patients and 15 caregivers refused to participate. Reasons for refusal included advanced age, fatigue, and other symptoms related to the heart problem. One-month outcome measures were obtained for 27 (14 in the EG and 13 in the CG) of the 32 dyads. Lost to follow-up were due to hospitalization (*n* = 3) or death (*n* = 2).

### Characteristics of the sample

The sample was composed of 32 patient-caregiver dyads. Baseline characteristics were similar in the EG and CG (see Table 2). The HF patients were primarily male, with a mean age of 67 years, retired, and living with a caregiver, all dyads except one were composed of spouses, the other dyad was composed of friends. The main clinical diagnosis at hospitalization was acute heart failure, arrhythmia/pacemaker problems, acute coronary syndrome, and other reasons (e.g., infection). Patients were hospitalized for a mean of 10 days and were prescribed a mean of 12 medications at hospital discharge.

**Table 2:** Socio-demographic and clinical characteristics of HF patients and caregivers

	EG (*N* = 16)	CG (*N* = 16)
	*n* (%)	
HF patient		
Sex, male	11 (69)	13 (81)
Age in years *mean (SD)*	67.8 (9.9)	66.9 (11.3)
Married or common law spouse	15 (94)	16 (100)
Not working	14 (88)	12 (75)
Education ≤ high school	11 (69)	9 (56)
Number of days of hospitalization *mean (SD)*	9.6 (5.7)	10.8 (9.0)
Reasons for hospitalization		
Heart failure	8 (50)	11 (69)
Pacemaker related problems/arrhythmia	3 (19)	2 (13)
Acute coronary syndrome	2 (13)	0 (0)
Other	3 (19)	3 (19)
New York Heart Association class		
I	1 (6)	0 (0)
II	7 (44)	6 (38)
III	7 (44)	8 (50)
IV	1 (6)	2 (13)
Mean percentage of ejection fraction *mean (SD)*	31.56 (14.7)	29.68 (10.4)
≥1 hospitalization in the last year (vs not)	5 (31)	6 (38)
≥1 emergency visit in the last year (vs not)	4 (25)	2 (13)
Number of medications at discharge mean (SD)	11.6 (2.9)	11.6 (4.5)
Caregiver		
Sex, male	6 (38)	3 (19)
Age in years mean (SD)	63.2 (11.4)	63.7 (10)
Education ≤ high school	10 (63)	9 (56)
Not working	11 (69)	11 (69)

### Feasibility results

Regarding the structure of the intervention, 12 of the 16 dyads received all five encounters of the planned interventions. The mean duration of the first two in-hospital encounters realized with all 16 dyad were 26 and 17 min, respectively. Some post-discharge planned telephone encounters were not feasible because the patients had been re-hospitalized. The mean duration for the first (*n* = 13), second (*n* = 14), and third (*n* = 13) telephone encounters was between 5 and 10 min. The five encounter was therefore delivered to 75 % of the dyads.

Results from the patient and caregiver intervention checklists are presented in Tables 3 and 4, respectively. Most patient checklist items were used in hospital encounter, and the frequency of use varied in the three post-discharge encounters. This was similar for the caregivers’ checklist items. Thus, the delivery of the intervention content was feasible, and each dyad received a different intervention.

**Table 3:** Assessment and nursing intervention checklist for the HF patient in the EG

	First in-hospital (*n* = 16)	After hospital discharge		
		1st week (*n* = 13)	2nd week (*n* = 14)	3rd week (*n* = 13)
Assessment (11 items)				
Questions about perceived competence				
How do you assess your ability to manage your heart failure symptoms?	100	92	100	92
What tells you that you have managed your heart failure well/badly?	94	85	93	85
Questions about autonomous motivation				
Describe your heart problem and the context of your first diagnosis.	100	8	0	0
What are you doing to manage the symptoms of heart failure?	100	92	100	92
Why are you doing this?	88	92	100	92
In your opinion, what caused your hospitalization?	94	54	64	39
How could you avoid another hospitalization, in your opinion?	88	69	71	62
Questions about relatedness				
What do you do when you need help?	100	100	100	100
What kind of help do you need most?	100	92	57	62
Does the help you receive correspond to your needs?	100	77	64	46
Do you receive help that you feel is useless? What?	82	54	64	31
Intervention (9 items)				
Discuss the impact of heart failure on the patient’s life.	100	69	36	23
Empathize with respect to the patient’s real-life limitations and complexity of his self-care practice.	93	85	86	100
Provide information to the patient on potential benefits of performing self-care activities.	93	77	79	92
Discuss actions taken by the patient towards his self-care practice.	100	100	100	100
If the patient expresses interest in practicing self-care, congratulate him on his choice and discuss with him the measures it will take to achieve his self-care goals.	100	92	100	100
If the patient expresses an interest in applying his self-care but stresses certain obstacles, suggest strategies. Example: In the case of thirst, sucking an ice cube instead of drinking water.	0	0	7	15
If the patient expresses his disinterest in practicing self-care, express respect for his current position and request permission to return to the issue later.	0	0	0	0
Discuss health behaviors successfully adopted (unique to heart failure or not) and provide positive reinforcement.	87	62	79	54
Normalize failures and invite the patient to think about their cause.	93	92	86	54

Data presented in percentage

**Table 4:** Nursing interventions checklist for the caregivers in the EG

	First in-hospital (*n* = 16)	After hospital discharge		
		1st week (*n* = 13)	2nd week (*n* = 14)	3rd week (*n* = 13)
Intervention (7 items)				
Follow-up of the previous encounter.	100	93	100	100
Explore the difficulties of adopting autonomy supportive behaviors.	100	100	100	100
Discuss various autonomy supportive behaviors.	100	100	100	100
Discuss strategies promoting the caregiver’s support.	100	93	85	85
Verbally explain autonomy supportive behaviors using examples from everyday life.	100	36	23	31
Using scenarios, verbally explain autonomy supportive behaviors.	100	7	0	8
Hand over the documents.	100	0	0	0

Data presented in percentage

### Acceptability results

Fourteen out of 16 patients in the EG responded to the TAPQ questionnaire. All of them found the intervention “very much” or “extremely appropriate” in helping them to manage their HF and would be willing to participate if a similar study was offered to them. All 14 HF patients were extremely satisfied based on the overall satisfaction question. For their 14 caregivers, results were similar except for their overall satisfaction and willingness to participate in a similar study. One out of the 14 caregivers indicated, he would “not at all” agree to participate in a similar study and one caregiver was only moderately satisfied with the intervention.

### Descriptive data on the outcome measures

As shown in Table 5, compared to the CG, the EG had better 1-month outcomes for self-care specific to HF (H2), internal extrinsic motivation (H4_c_), intrinsic motivation (H4_d_), and caregiver’s feeling that they provide higher level of support (H6) (all reflected by higher scores). They also had better outcomes on A-motivation (H4_a_) (reflected by lower scores). On the other hand, the EG had worse outcomes (reflected by higher scores) on external extrinsic motivation (H4_b_). Mean scores were identical for H1, H3, and H5 outcomes. Finally, the EG obtained a mean score higher than 70 on the SCHFI scale (H2) indicating adequate self-care activities [21], whereas the mean score for the control group was below 70.

**Table 5:** Mean scores for outcome measures at baseline and 1 month

	EG mean (SD)	CG mean (SD)
H1: General self-care management^a^		
Baseline	46.19 (13.13)	46.31 (8.97)
1 month	50.57 (4.89)	50.76 (6.73)
H2: Self-care specific to HF^a^		
Baseline	49.37 (20.37)	46.25 (14.50)
1 month	75.23 (10.60)	69.48 (16.66)
H3: Perceived competence to manage HF^a^		
Baseline	20.13 (4.08)	19.06 (5.30)
1 month	23.29 (3.27)	23.85 (4.02)
H4: Behavioral Questionnaire of the Elderly-Health activities		
H4a A-motivation^b^		
Baseline	7.88 (3.98)	5.87 (3.46)
1 month	4.14 (2.18)	6.08 (3.64)
H4b: External extrinsic motivation^b^		
Baseline	13.00 (4.77)	15.13 (4.73)
1 month	16.29 (6.40)	13.85 (5.32)
H4c: Internal extrinsic motivation^a^		
Baseline	16.65 (2.93)	16.38 (3.16)
1 month	19.29 (1.77)	17.54 (3.23)
H4d: Intrinsic motivation^a^		
Baseline	11.13 (5.58)	9.63 (4.26)
1 month	10.86 (7.55)	9.77 (5.48)
H5: Family Care Climate Questionnaire-Patient version^a^		
Baseline	57.56 (7.97)	60.19 (5.24)
1 month	61.86 (6.63)	62.46 (6.57)
H6: Family Care Climate Questionnaire-Family version^a^		
Baseline	58.13 (4.94)	57.94 (6.69)
1 month	60.21 (4.93)	57.85 (6.45)

*N* = 16 both for the EG and CG for the baseline scores and *N* = 14 (EG) and 13 (CG) for the 1-month scores because five patients were hospitalized or dead

^a^Higher scores represent better outcomes

^b^Lower scores represent better outcomes

## Discussion

The present study demonstrates that this intervention was feasible and acceptable in terms of its structure and content. For instance, all 16 EG dyads received the first and second planned encounters during hospitalization and 12 dyads out of the 16 EG dyads received all three planned post-discharge telephone encounters. Thus, three quarters of the dyads received all five planned encounters. Both hospital encounters were shorter than the planned 45 min, and their content corresponded adequately to what was planned. However, in terms of recruitment, many patients were not eligible because they were either followed by a specialized heart failure clinic or did not have a caregiver living with them. Requiring a live-in caregiver to participate in the intervention was a challenge in terms of designing this trial and will require alternative strategies for a larger trial. For instance, could caregivers who do not live with the patient be included? A similar intervention could also be suggested for patients with no caregiver. While still based on the SDT framework, this intervention would focus on strengthening the patient’s perceived competence and intrinsic motivation rather than on “relatedness” as in the present study. Recruitment was also hampered by a number of refusals among eligible patients. Reasons for refusal included advanced age, fatigue, and other symptoms related to the heart problem.

The potential effectiveness of the intervention is promising, with the largest differences favoring the EG for H2, self-care activities specific to HF, and for H4_a_ A-motivation. This is especially meaningful since A-motivation is a barrier for behavioral changes because patients lack motivation to practice self-care and see no link between poor self-care (e.g., fluid restriction) and consequences (e.g., cardiac decompensation). On the opposite side of this continuum, higher intrinsic motivation is thought to enhance long-term adherence to self-care activities. One result, for external extrinsic motivation (H4_b_), was opposite of what was expected, favoring the CG. Therefore, future studies could consider retaining self-care activities as the primary outcome, especially for the potential impact on clinical outcomes such as HF decompensation and rehospitalization.

In the last decade, the literature on challenges related to delivering personalized and complex interventions has grown partly because a non-standardized intervention is more difficult to replicate than a tailored one [15]. The present experimental intervention includes patient and caregiver intervention checklists. By providing a precise description of the exact intervention content and structure, these checklists can facilitate replication in future studies. These checklists also served as a guide to respect the fundamental principles of the Self-Determination Theory.

The learning activities included in the intervention aimed at enhancing caregiver-patient interaction during hospitalization were also transferable after discharge, providing the dyads with useful strategies and tools for when they return home. For instance, given that the patient’s diet and liquid restrictions may generate some family conflict post-discharge, role-playing between caregivers and patients may be useful in anticipating and “practicing” alternative strategies.

A challenge in the present study was to identify specific scales to assess autonomous motivation because most studies base their outcome measures on quality of life, mortality, and self-care. The present study used an open-access scale (Behavioral Questionnaire of the Elderly-Health subscale [22]), available in French and English, that is specifically based on SDT, but not often used in the HF literature. This was a novel opportunity to widen our understanding of adherence to self-care activities in HF patients. In addition, as suggested by Riegel and colleagues [21], low Cronbach alpha values on the SCHFI in the present study, as well as a previous study [21], may be explained by the lack of theoretical correlation between different self-care items, for instance, medication adherence and routine exercise.

### Study limitations

The present study had some limitations. The first limitation is the sample size, while adequate for a pilot study did not permit hypothesis testing [24]. Single-center recruitment is the second limitation because the findings cannot be generalized beyond a tertiary cardiology center. Other methodological choices present further limitations, such as the fact that the intervention is administered by a single nurse limits transferability and that self-reported measures may be a source of bias in the study results.

## Conclusions

Pilot studies are now recognized as a valuable, if not vital step in evaluating the feasibility and acceptability of nursing interventions to be tested in future clinical trials [24]. The use of a strong theoretical frameworks to design and test nursing intervention studies is recommended to develop coherent knowledge. The scientific community agrees that future interventions in HF must involve caregivers and include education and counseling [2]. Aside from recruitment challenges, the experimental intervention, inspired by Self Determination Theory, tested in this study was feasible and acceptable and its potential effectiveness shows great potential as all but one result favored the EG. The present study adds to the literature by offering avenues to propose specific learning activities for caregivers and focusing on enhancing patient autonomy in self-care practices. This approach is in line with clinical guidelines highlighting the need to provide knowledge and skills, as well as abilities and motivation to perform self-care [4]. As this study presents preliminary findings in the context of the pilot study, this intervention should be tested in a larger trial.

## Abbreviations

CG, control group; EG, experimental group; FCCQ-F, Family Care Climate Questionnaire-Family version; FCCQ-P, Family Care Climate Questionnaire-Patient version; HF, heart failure; PCS, Perceived Competence Scale; SCHFI, Self-Care of Heart Failure Index; SDT, Self-Determination Theory; TAPQ, treatment acceptability and preference questionnaire
